# Assessing male and female clinicians’ intentions for a third child in China: A cross-sectional survey analysis with gender-specific insights

**DOI:** 10.7189/jogh.15.04001

**Published:** 2025-01-03

**Authors:** Dandan Zhang, Fen Liu, Tianxin Cui, Xinqi Zhuang, Jianzhong Zhang, Xiaoyu Lei, Yin-Ping Zhang

**Affiliations:** 1 School of Nursing, Xi’an Jiaotong University Health Science Center, Xi’an, Shaanxi Province, China; 2 Institute of Clinical Research, Affiliated Nanhua Hospital, Hengyang Medical School, University of South China, Hengyang, Hunan Province, China; 3 Department of Gynecology and Obstetrics, The First Affiliated Hospital, Hengyang Medical School, University of South China, Hengyang, Hunan Province, China; 4 School of Health in Social Science, University of Edinburgh, Edinburgh, Scotland, UK

## Abstract

**Background:**

As fertility rates decline and population ageing intensifies, the conflict between career and childbearing continues to impact clinicians, especially women. Exploring gender differences in the fertility intentions of male and female clinicians could help with identifying barriers to childbearing, developing effective policies to support work-life balance, and addressing the gap in research on gender disparities in this field.

**Methods:**

We conducted a cross-sectional survey among health care personnel in Chinese public hospitals. Through cluster sampling from highly active WeChat groups, we gathered 698 responses from clinicians to the third fertility intention questionnaire online. We then used descriptive statistics and χ^2^ tests for analysis.

**Results:**

Men (28.28%) had higher intentions of having a third child than women (20.71%) (*P* = 0.013). In terms of reasons, female clinicians were more concerned than male clinicians about the impact on their career development (*P* = 0.002), difficulties in job hunting (*P* = 0.039), and physical injuries caused by multiple births (*P* < 0.001), and whether the elderly can help (*P* = 0.001). Conversely, men’s apprehensions centred on economic factors such as real house costs (*P* < 0.001), policy support (*P* = 0.036), and wives’ disagreement (*P* < 0.001). In discussing governmental interventions, men showed a higher level of interest in policies related to child care (*P* < 0.001), employment stability for women (*P* < 0.001), extended maternity leave (*P* < 0.001), and financial assistance than women (*P* < 0.001).

**Conclusions:**

Our findings show substantial gender-specific differences in third-child fertility intentions among clinicians. To address this, the government should consider divisions in family roles, future societal needs, and women’s career development. Policies should focus on balancing work and family by offering affordable childcare, flexible parenting leave, financial incentives, and career support, ensuring childbirth does not negatively impact women’s professional growth, and fostering gender equality in parenting.

The global fertility rate has significantly decreased in the past 50 years, with the world population growth rate dropping to below 1% by 2020 [1]. This is especially true for countries in Asia and Africa, where the rates in Japan and South Korea, for example, dropped below the replacement level of 2.1 children per woman [2]. Conversely, countries like Niger and Angola in Africa have maintained higher fertility rates exceeding five children per woman [2]. To promote the sustainable development of the population, economy, and society, and to actively respond to the phenomenon of population ageing, the Chinese government has continuously been adjusting its fertility policy since 2011 [3,4]. Succeeding the previous selective two-child and universal two-child policies [5], the ‘three-child’ policy for couples was introduced in 2021 [6], signalling the government’s resolve to promote higher fertility levels. Yet the success of such efforts depends on other, related national policies and the intentions of the population of childbearing age towards fertility. For example, statistics show that, despite these policy changes, the average child-bearing fertility rate in China remains low at 1.2 [7,8], even lower than Japan’s fertility rate of 1.3 [8,9]. The country is simultaneously confronted with the issues of population decline and rapid ageing, especially as the gradual liberalisation of fertility policies outlined above did not lead to a sustained increase in births. China’s population saw a decline in 2022 [10] due to low fertility intention and postponement in childbearing [9–12]. These trends led to discussions in China on how to tackle these challenges, improve the population’s fertility intention, and address any related influencing factors.

Studies on fertility intentions primarily focussed on women of childbearing age and their spouses [12,13], along with specific groups such as adolescents [14], women with disabilities [15], AIDS patients [16], cancer patients [17], and others. Discussions on revising laws and policies, meanwhile, have focussed on actively implementing measures that support the ‘three-child’ policy, particularly in areas of infant care, education, taxation, and housing, as well as proposals for providing cash incentives to couples who have an additional child [18].

As a highly educated and responsible professional group, clinical physicians’ fertility decisions reflect their individual choices and significantly influence personnel allocation within the healthcare system. Therefore, better understanding the challenges they face in balancing career development and fertility is particularly important. Surveys have shown occupational differences in intentions towards having a second child, indicating that the fertility intentions of medical staff are higher than those of state-owned enterprise employees [19,20]. A survey [21] on the fertility intention of married female clinicians showed that 27.1% were willing to have a second child, while 68.6% were reluctant due to concerns about reduced work efficiency and negative impacts on career advancement. Another study among doctors and nurses in China found that 46.7% expressed a desire to have a second child, while 18.5% wanted to have a third child [22]. Previous studies have identified educational attainment as a crucial factor influencing individuals’ fertility intentions, whereby women with higher education levels typically exhibited lower fertility intentions [23,24]. Yang et al. [25] also found that the longer the postgraduate training period for female clinicians, the lower their willingness to have children.

The prerequisite of having children is that the couple has the same fertility intention rather than the unilateral decision of the husband or wife [26]. Differences in individual characteristics, their own upbringing, and the social division of labour frequently lead to men and women having distinct intentions and attitudes towards fertility [27,28]. In China, fathers traditionally bear more responsibility for supporting the family, making income and housing ownership, which are significant factors affecting their fertility intention; mothers, in turn, are more prominently involved in childcare and experience greater stress when facing conflicts between family and work [29,30]. Yet as women achieve relative independence in career advancement and economic income, the traditional family division of labour is gradually changing, which, in turn, significantly affects women's career development [31,32] and challenges their perception of fertility, especially in high-income and high-education groups [21,31]. Clinical physicians in China face a high-pressure, intense work environment, further exacerbating the conflict between career development and fertility [25]. Studies have shown that female clinicians, especially female surgeons, are more likely to postpone childbirth to prioritise their careers until professional training is completed, as well as that they often use reproductive technology [33,34]. The main factors for being unwilling to bear a second child among the general population are family income, career development, and child care [20,35]. Other studies have identified age, educational levels, and income as the main factors affecting male clinicians' fertility intention [36].

By studying gender differences in clinicians' fertility intentions, we can better understand how male and female clinicians approach family planning and the factors influencing their decisions and identify potential barriers and challenges that may prevent them from expanding their families. This information could then inform the development of interventions and policies to address these issues. Such research could also help us explore the experiences of diverse groups of clinicians and identify obstacles to diversity and inclusion in health care. With this in mind, we wanted to explore the differences in the intention of male and female clinicians towards having a third child, compare the underlying reasons for their perspectives, and provide targeted recommendations from a gender perspective.

## METHODS

### Study design

This was a cross-sectional survey of clinicians in Chinese public hospitals, conducted from December 2021 to January 2022.

### Sample and setting

We included clinicians aged 20–49 years who worked in public hospitals and voluntarily consented to participate. We excluded non-Chinese clinicians and those already pregnant with or having a third child.

We calculated our sample size per the following formula:



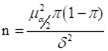



where *μ_α/2_*  = 1.96, δ = 0.03, and π = 0.18 [22]. This resulted in a suggested sample size of 644 clinicians. Here we derived the sample size of clinicians from a large-scale cross-sectional survey we conducted previously (manuscript submitted for publication elsewhere). This survey encompassed healthcare professionals from various specialties and backgrounds across the country, examining their willingness to have a third child.

Through cluster sampling, we randomly selected 30 highly-active WeChat groups primarily used for academic exchange among health care professionals nationwide. Thematically, 15 groups covered various specialties in clinical departments, such as internal medicine, surgery, obstetrics and gynaecology, paediatrics, infectious diseases, and others; five covered topics such as hospital management, medical quality, hospital infection control; and the remaining 10 groups discussed outpatient services, nursing, pharmacy, traditional Chinese medicine, rehabilitation, interventional therapy, radiology, ultrasound, and topics related to medical auxiliary departments and community health care services. In terms of size, these groups consisted of approximately 15 000 health care workers with various educational backgrounds, experiences, and ages to ensure the representativeness of the sample.

In total, 705 clinicians completed our questionnaire; after we screened their demographics based on our inclusion and exclusion criteria, we included 698 participants in this survey.

As a sub-study of the aforementioned large-scale study project, we focussed on the third childbirth intentions of clinicians and conducts an in-depth analysis of the gender differences between male and female clinicians in this regard.

### Questionnaire and study variables

The study questionnaire was prepared based on an extensive literature review and drew upon Zhang and colleagues’ survey among the reproductive-aged population on intentions of having a third childbirth [13]. The questionnaire had 37 questions on sociodemographic information and fertility intentions. Before the formal survey, we conducted two pilot tests of the questionnaire among a total of 46 healthcare workers, to ensure the clarity and comprehensibility of the questions. We did not include these data in this analysis.

Sixteen items collected sociodemographic data such as gender, age, ethnicity, family monthly income, education, department, professional title and position, house ownership, marital status, family structure, previous childbearing experience, and others. The items of previous childbearing experience were only completed by subjects who had given birth to children, and the system automatically skipped these questions when the subjects choose ‘have not given birth’.

The second part of the questionnaire contained 21 items with 19 multiple-choice questions and two open-ended questions. They covered five dimensions: fertility intentions (one item), attitude to parenting (six items), intentional or unintentional reasons for the third birth (eight items), professional attitude (four items), and attitude to the ‘three-child’ policy (two items). The overall reliability of the questionnaire, as measured by Cronbach’s α, was 0.79. The content validity index established through expert scoring was 0.87, the internal consistency reliability coefficients for various dimensions ranged from 0.73 to 0.91, and the test-retest reliability coefficients ranged from 0.463 to 0.742. The questionnaire employed in this study showed good reliability and validity.

### Data collection

We posted a survey questionnaire on Sojump [37], the most popular online platform in China, and then automatically generated e-posters with QR codes and fast survey links, which we then published in the selected WeChat groups from which we recruited our participants. After scanning the QR code on the e-poster or clicking on the link, the participants were explained the purpose of the study, that their participation was anonymous and voluntary, and were asked to provide informed consent before filling out the questionnaire. Each participant was assigned a unique identification number to avoid repeated submissions. Participants were also required to answer all survey items before submission, ensuring they finished the questionnaire completely and accurately. The online system containing the questionnaire automatically recorded its completion status and completion time for each participant; questionnaires completed in less than two minutes were considered invalid. Two quality control members regularly inspected the completed questionnaires to ensure the integrity and accuracy of the recorded data. Research team members confirmed the quality control of the collected data again after the collection: this included analysts and quality control members collectively checking the data for for errors, inconsistencies, missing information, validity, accuracy, and consistency. The analysts also performed statistical analyses and data mining to validate the reliability and effectiveness of the data.

### Statistical analysis

We used descriptive analysis to summarise the demographic data, third childbirth intention, and reasons, with the continuous data presented as means and standard deviations (SDs) and categorical data as frequencies and percentages. We used χ^2^ tests to compare the gender differences in fertility intentions between male and female clinicians. Two-sided *P*-values <0.05 indicated statistical significance. All data were analysed in SPSS, version 18.0 (IBM, New York, USA).

## RESULTS

### Demographic data

We included responses from 698 clinicians, comprising 309 females (44.27%) with a mean age of 32.85 years (SD = 5.32) and 389 males (55.73%) with a mean age of 36.11 years (SD = 5.78). Most of them were married (female: 79.94%; male: 85.35%) and of Han ethnicity (female: 86.73%; male: 76.61%). Most participants had one or two children (female: 76.05%, male: 80.72%) and had loans or a fully paid housing (female: 79.94%; male: 88.95%) (**Table 1**).

**Table 1:** Socio-demographic data*

Variables	Female (n = 309)	Male (n = 389)
Age in years, x̄ (SD)	32.85 (5.32)	36.11 (5.78)
Marital status		
*Unmarried*	62 (20.06)	57 (14.65)
*Married*	247 (79.94)	332 (85.35)
Ethnic group		
*Han nationality*	268 (86.73)	298 (76.61)
*Minority*	41 (13.27)	91 (23.39)
Education		
Diploma/associate degree	6 (1.94)	4 (1.03)
*Bachelor’s degree*	100 (32.36)	85 (21.85)
*Master’s degree*	169 (54.69)	187 (48.07)
*Doctor’s degree*	34 (11.00)	113 (29.05)
Family monthly income		
*≤RMB 10 000 (USD 1406)*	97 (31.39)	49 (12.60)
*RMB 10 001–20 000 (USD 1407–2813)*	119 (38.51)	125 (32.13)
*RMB 20 001–29 999 (USD 2813–4219)*	66 (21.36)	145 (37.28)
*≥RMB 30 000 (USD 4219)*	27 (8.74)	70 (17.99)
Professional title		
*Primary*	75 (24.27)	70 (17.99)
*Intermediate*	145 (46.93)	156 (40.10)
*Senior*	71 (22.98)	152 (39.07)
*None*	18 (5.83)	11 (2.83)
Position		
*Senior manager*	32 (10.36)	75 (19.28)
*Middle manager*	84 (27.18)	129 (33.16)
*Ordinary employee*	176 (56.96)	174 (44.73)
*Temporary worker*	17 (5.50)	11 (2.83)
House ownership		
*Housing without loan*	79 (25.57)	127 (32.65)
*Housing with loan*	168 (54.37)	219 (56.30)
*Tenancy*	43 (13.92)	25 (6.43)
*Live with parents' house*	14 (4.53)	14 (3.60)
*Dormitory*	5 (1.62)	4 (1.03)
Family structure (only for married)		
*Both are one-child families*	49 (19.84)	48 (14.24)
*One of the partners is a one-child family*	107 (43.32)	141 (41.84)
*Both are two or more child families*	91 (36.84)	143 (42.43)
Number of existing children		
*None*	74 (23.95)	75 (19.28)
*One*	129 (41.75)	149 (38.30)
*Two*	106 (34.30)	165 (42.42)
Gender of existing child		
*Boy*	78 (33.19)	96 (30.57)
*Girl*	95 (40.43)	124 (39.49)
*Both*	62 (26.38)	94 (29.94)

SD – standard deviation, x̄ – mean

*Presented as n (%) unless specified otherwise.

### Comparison of fertility intentions between male and female clinicians

About one-quarter of the clinicians (24.93%) indicated that they strongly intended/intended to have a third child, and the intention of male clinicians to have a third child accounted for 28.28%, which was significantly higher than that of female clinicians (20.71%) (*P* = 0.013) (**Table 2**).

**Table 2:** Comparison of fertility intentions to have a third child between male and female clinicians

Variables	Total (n = 698), n (%)	Female (n = 309), n (%)	Male (n = 389), n (%)	*χ^2^*	*P*-value
Strongly intended	46 (6.59)	21 (6.80)	25 (6.43)	10.876	0.013
Intended	128 (18.34)	43 (13.91)	85 (21.85)		
Uncertain	99 (14.18)	38 (12.30)	61 (15.68)		
Unintended	425 (60.89)	207 (66.99)	218 (56.04)		

### Reasons for not intending to have a third child

**Figure 1:**
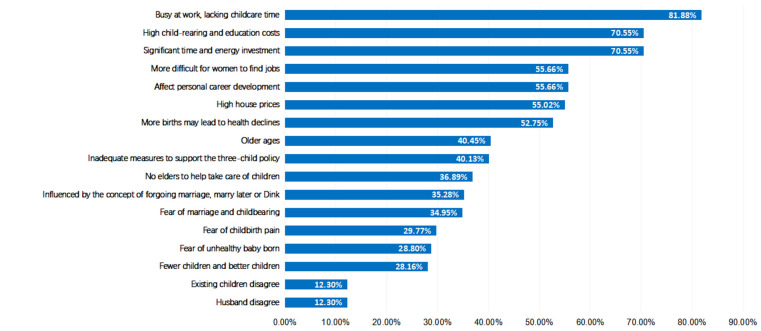
Reasons for female clinicians not intending to have a third child.

The main reasons why female clinicians did not intend to have a third child were being too busy at work and lacking childcare time (81.88%), high child-rearing and education costs (70.55%), significant time and energy investment (70.55%), negative impacts on job prospects and career development (55.66%), and perceiving that more births may lead to health declines (52.75%) (**Figure 1**).

**Figure 2:**
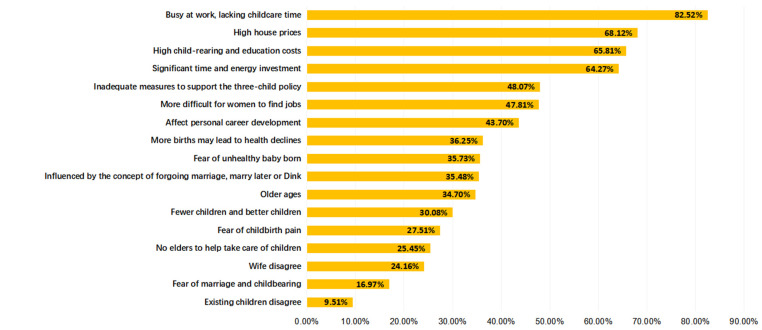
Reasons for male clinicians not intending to have a third child.

The main reasons why male clinicians did not intend to have a third child are being too busy with work and lacking childcare time (82.52%), high housing prices (68.12%), high child-rearing and education costs (65.81%), significant time and energy investment (64.27%), and inadequate measures to support the three-child policy (48.07%) (**Figure 2**).

The comparison of reasons why males and females did not intend to have a third child showed that the latter were more concerned than the former about the impact on their career development (*P* = 0.002), difficulties in job hunting (*P* = 0.039), and the likelihood that having more births will lead to decreased health conditions (*P* < 0.001). They also worried more about the lack of elder support in taking care of the children (*P* = 0.001) and were more prone to fear marriage and childbirth (*P* < 0.001). Meanwhile, male clinicians were more concerned about high housing prices (*P* < 0.001), inadequate measures to support the three-child policy (*P* = 0.036), and disapproval of their wives (*P* < 0.001) (**Table 3**).

**Table 3:** Comparison of reasons for clinicians did not intend to have a third child

Reasons	Female (n = 309), n (%)	Male (n = 389), n (%)	*χ^2^*	*P*-value
Busy at work, lacking childcare time	253 (81.88)	321 (82.52)	0.171	0.680
Significant time and energy investment	218 (70.55)	250 (64.27)	3.007	0.079
High child-rearing and education costs	218 (70.55)	256 (65.81)	1.776	0.183
Affects personal career development	172 (55.66)	170 (43.70)	9.860	0.002
More difficult for women to find jobs	172 (55.66)	186 (47.81)	4.246	0.039
High house prices	170 (55.02)	265 (68.12)	12.599	<0.001
More births may lead to health declines	163 (52.75)	141 (36.25)	19.080	<0.001
Inadequate measures to support the three-child policy	124 (40.13)	187 (48.07)	4.398	0.036
No elders to help take care of children	114 (36.89)	99 (25.45)	10.635	0.001
Influenced by the concept of forgoing marriage, late marriage, or dual income/no kids	109 (35.28)	138 (35.48)	0.003	0.956
Fear of marriage and childbearing	108 (34.95)	66 (16.97)	29.764	<0.001
Older age	125 (40.45)	135 (34.70)	2.435	0.119
Fear of childbirth pain	92 (29.77)	107 (27.51)	0.434	0.510
Fear of unhealthy babies born	89 (28.80)	139 (35.73)	3.760	0.052
Fewer children and better children	87 (28.16)	117 (30.08)	0.307	0.579
Husband/wife disagree	38 (12.30)	94 (24.16)	15.814	<0.001
Existing children disagree	38 (12.30)	37 (9.51)	1.394	0.238

### Comparison of clinicians' recommendations of supporting measures for the birth of the third child

Regarding the recommendations of the government's support measures for third-child birth, male clinicians were more active in providing childcare services (*P* < 0.001), ensuring female employment (*P* < 0.001), extending maternity leave (*P* < 0.001), providing financial subsidies (*P* < 0.001), and supporting rectifying bad marriage customs, and high bride prices than female clinicians (*P* < 0.001) (**Table 4**).

**Table 4:** Comparison of clinicians' recommendations of supporting measures for the birth of the third child

Recommendations	Female (n = 309), n (%)	Male (n = 389), n (%)	*χ^2^*	*P*-value
Affordable and high-quality child care and kindergarten services	194 (49.87)	249 (80.58)	70.05	<0.001
Equitable access to high-quality education	252 (81.55)	337 (86.63)	3.371	0.066
Guaranteed employment for women	218 (56.04)	252 (81.55)	50.96	<0.001
Extension of maternity leave	189 (48.59)	202 (65.37)	19.695	<0.001
Cash, tax cuts, subsidies, concessions, and other benefits	219 (56.30)	260 (84.14)	62.009	<0.001
Rectify bad marriage customs, high-priced bride prices, etc.	110 (28.28)	156 (50.49)	36.009	<0.001

## DISCUSSION

This cross-sectional survey analysed gender differences in fertility intentions among 698 clinicians from several public hospitals in China and found that 24.93% of clinicians expressed a desire to have a third child. This is slightly lower than the percentage previously observed among health care workers in regards to having a second child (29.10%) [25,38]. Specifically, 28.28% of male clinicians wanted a third child – a proportion significantly higher than the 20.71% observed for female clinicians (*P* = 0.013). Primary factors deterring male clinicians from their intention to have a third child included high house prices, support policies, and the disapproval of their wives. In contrast, female clinicians were more concerned about career obstacles, physical damage caused by multiple births, and the availability of elderly help for childcare. These findings suggest that the government urgently needs to provide support, care, and preferential guarantee systems to solve or improve related problems among clinicians.

In a survey of 15 332 childbearing-age participants in China, Zhang et al. [13] found that men were more than twice as willing to have three children compared to women. Odusina et al. [39] also observed that men showed a higher desire for more children than their wives, which is similar to our findings. A previous study has indicated that women’s fertility desire peaks as they approach the ideal childbearing age [40]. In our study, female clinicians within this age range reported often experienceing significant stress due to academic or clinical responsibilities, potentially delaying their childbearing plans. Testa et al. [41] observed that in families with two children, the partner desiring a second child held more decision-making power. However, the decision to continue having children after the second child is predominantly influenced by personal preference. If one partner in the family is opposed to the idea or if other obstacles arise, the desire for another child may be more difficult to pursue [41]. Our study similarly focussed on the intention to have a third child, which may also depend on personal preference and external factors.

Ning et al. [42] observed a decrease in intensions to have a third child as education level increased. However, our study showed a contrasting trend, with a high percentage of female (65.69%) and male clinicians (77.12%) possessing advanced master's or doctorate degrees and expressing a stronger desire for a third child compared to the general population (9.6% [42] and 12.2% [13] in two other studies). This may be related to the relatively high economic income of clinicians. Zhu et al. [43] showed that, among families with two children, parents with family financial difficulties and children's educational barriers are more likely to lack the intention of having a third child. Kang et al. [44] surveyed 4406 Chinese parents with children after implementing the new three-child policy and found that people with an annual family income of more than RMB 120000 (USD 16 862) were even less worried about childcare costs. In our study, 68.61% of female and 87.40% of male clinicians had an annual family income exceeding RMB 120 000 (USD 16 862). Nevertheless, 70.55% of female clinicians and 65.81% of male clinicians were still worried about the high cost of raising children and education. Chudnovskaya et al. [45] also observed that higher income and social class background were positively correlated with father status, and the correlation did not change with time. However, there is no positive correlation between educational prestige (higher or traditional university degrees) and paternity [45].

Women's capability in family decision-making often depends on their age, education, professional status, and income [46]. Female clinicians have higher degrees, professional levels, and incomes, but may miss the best childbearing period. Thus, they are more likely to prioritise childbearing within the first five years post-graduation. The career development of female clinicians will also be affected by considerations of their fertility [46–48]. However, male clinicians are less affected by fertility and are more likely to work hard to obtain higher incomes and career development [49,50]. They have greater flexibility in making fertility decisions, allowing them to make choices that do not disrupt the ideal fertility period. In our study, female clinicians were more concerned about the impact on job search or career development than male clinicians (*P* = 0.002), while male clinicians were more likely to have no intention of having a third child due to their wives' disagreement (*P* < 0.001).

The research by Zhou et al. [51] showed that the overtime hours of men are significantly positively correlated with fertility intention, while women's housework time and nursing time are significantly positively correlated with fertility intention. This gender-based division of labour will likely lead to ‘work-life conflict’ at the individual level, but results in ‘work-life balance’ at the family level; however, this balance comes at the expense of women's development, leading to a lower fertility desire among young women compared to men [51]. Therefore, the government should protect the legitimate right of workers to have more time to return to their families, especially men [51]. In South Korea, fathers residing in households where both parents are employed and who take parental leave significantly contribute to housework and childcare, but their willingness to have a second child is lower and conflicts between their work and their family increase significantly [52]. The main reasons for not intending to have a third child in this study also included that female clinicians were worried about childcare time, the costs of their own energy, and the lack of help from the elderly in childcare. Aside from worrying about the time cost of taking care of children, male clinicians in our study also felt the pressure of high housing prices and hoped to have better-supporting policies to promote fertility. Therefore, to promote fertility, the government should fully consider gender differences in fertility intentions, role divisions, the future development of society, and the needs of women's career development when formulating support measures, and ensure to maintain a balance between work, family, and life.

This study has some limitations. First, we used a convenience sampling method to recruit participants through WeChat groups, which might have lead to selection bias. Moreover, while our sample is drawn from 34 provinces throughout China and covers participants of different sociodemographic backgrounds, it is worth noting that 70% of the participants came from southern and central regions of China. Previous studies have shown that intentions toward fertility rates may vary by region [13,53], but we were unable to consider this fully in our analysis, leaving a gap for future studies.

## CONCLUSIONS

Approximately one-fourth of clinicians in our study planned to have a third child, with male clinicians showing a higher inclination than females. However, we observed a disparity between the genders in further analyses, as female clinicians expressed concerns for challenges that birth might pose for their career, the physical toll of multiple pregnancies, and the availability of elderly support. Male clinicians were more concerned with economic factors such as housing costs, policy support, and spousal disagreement.

These findings highlight significant gender-specific differences in clinicians’ intentions to have a third child. In response, policymakers should adopt a nuanced, gender-informed perspective in crafting pronatalist strategies. Support measures should consider gender differences, role divisions, societal development, and women’s career needs to balance work, family, and life. To prevent women from being disadvantaged in their careers due to childbirth and to enhance gender equality in parenting, these measures should include affordable childcare services, flexible leave and work arrangements, financial incentives, and support for women’s career advancement.
